# Age-Triggered and Dark-Induced Leaf Senescence Require the bHLH Transcription Factors PIF3, 4, and 5

**DOI:** 10.1093/mp/ssu109

**Published:** 2014-10-08

**Authors:** Yi Song, Chuangwei Yang, Shan Gao, Wei Zhang, Lin Li, Benke Kuai

**Affiliations:** ^a^State Key Laboratory of Genetic Engineering, Institute of Plant Biology, School of Life Sciences, Fudan University, Shanghai 200433, People’s Republic of China; ^b^National Key Laboratory of Plant Molecular Genetics and National Center for Plant Gene Research (Shanghai), Institute of Plant Physiology and Ecology, Shanghai Institutes for Biological Sciences, Chinese Academy of Sciences, Shanghai 200032, People’s Republic of China

**Keywords:** phytochrome-interacting factor (PIF), leaf senescence, chloroplast deterioration, NYE1/SGR1, GLK2, ethylene.

## Abstract

Leaf senescence can be triggered and promoted by a large number of developmental and environmental factors. Numerous lines of evidence have suggested an involvement of phytochromes in the regulation of leaf senescence, but the related signaling pathways and physiological mechanisms are poorly understood. In this study, we initially identified phytochrome-interacting factors (PIFs) 3, 4, and 5 as putative mediators of leaf senescence. Mutations of the PIF genes resulted in a significantly enhanced leaf longevity in age-triggered and dark-induced senescence, whereas overexpressions of these genes accelerated age-triggered and dark-induced senescence in *Arabidopsis*. Consistently, loss-of-function of PIF4 attenuated dark-induced transcriptional changes associated with chloroplast deterioration and reactive oxygen species (ROS) generation. ChIP–PCR and Dual-Luciferase assays demonstrated that PIF4 can activate chlorophyll degradation regulatory gene *NYE1* and repress chloroplast activity maintainer gene *GLK2* by binding to their promoter regions. Finally, dark-induced ethylene biosynthesis and ethylene-induced senescence were both dampened in *pif4*, suggesting the involvement of PIF4 in both ethylene biosynthesis and signaling pathway. Our study provides evidence that PIF3, 4, and 5 are novel positive senescence mediators and gains an insight into the mechanism of light signaling involved in the regulation of leaf senescence.

## SUMMARY

Numerous lines of evidence have suggested an involvement of phytochromes in the regulation of leaf senescence, but the related signaling pathway and physiological mechanisms are poorly understood. In this study, we demonstrated that PIF3, 4, and 5, the master regulators of light signaling, modulate age-triggered and dark-induced senescence and particularly PIF4 positively regulates leaf senescence by directly targeting genes related to chlorophyll degradation and chloroplast activity maintaining as well as ethylene biosynthesis.

## INTRODUCTION

Leaf senescence is a developmentally programmed event, which involves a number of physiological, biochemical, and molecular changes, including a decline in photosynthetic efficiency, decreases in chlorophyll and protein contents, and increases in membrane ion leakage and expression of senescence-associated genes (SAGs) (Sarwat et al., 2013). The chloroplast is the organelle which shows the earliest sign of senescence symptom (Soudry et al., 2005). How a functional chloroplast initiates its senescence process is not precisely known yet. What is known is that ORE1 (ANAC092), a NAC transcription factor (TF) known as a positive regulator of senescence (Kim et al., 2009), antagonizes the transcriptional activity of GLK1/GLK2 (Golden 2-like Transcription factor 1/2), the crucial chloroplast development promoters and activity maintainers (Waters et al., 2009), by interacting with them at protein level. This antagonizing interaction triggers a shift from chloroplast maintenance towards chloroplast deterioration (Rauf et al., 2013). Moreover, chlorophyll degradation regulator NYE1 (Non-yellowing 1, also named SGR1) recruits several chlorophyll catabolic enzymes (CCEs) to promote chlorophyll degradation during leaf senescence (Ren et al., 2007; Sakuraba et al., 2012).

Ethylene is known as an endogenous inducer of leaf senescence as well as fruit ripening and flowering (Bleecker and Kende, 2000), and increased expression of numerous genes encoding ethylene biosynthesis were detected in senescing leaves (Breeze et al., 2011). However, exogenous ethylene only can induce senescence in the leaves at a defined age (Jing et al., 2005), indicating that some developmental stage-associated factors are synergistically involved in the ethylene induction of senescence. The trifurcate feed-forward regulation of age-triggered senescence involving *EIN2*, *EIN3*, and *ORE1* is identified as an important ethylene-related regulation pathway of senescence (Kim et al., 2009).

Darkness, as an extreme light condition, is often used to induce rapid and synchronous senescence in detached leaves (Weaver and Amasino, 2001). A dark-detached system has been widely used as a senescence model to study the age-triggered senescence. Many functional SAGs, such as *ORE9* (Woo et al., 2001), *NAC029* (Guo and Gan, 2006), and *NAC092* (Kim et al., 2009), have been found to function in both age-triggered and dark-induced programs, suggesting a partially shared mechanism between them. However, compared to synchronized leaf senescence induced by dark, age-triggered leaf senescence characteristically displays a gradient of senescence from the tip to the base. Transcriptome profiling analysis indicated that the senescence program occurring in developmentally senescing leaves is significantly different from that in detached dark-held leaves (Buchanan-Wollaston et al., 2005; Breeze et al., 2011).

Involvement of light in regulating leaf senescence has been indicated in a number of studies. Red light inhibits the loss of chlorophyll, which can be reversed by subsequent illumination with far-red light (De Greef et al., 1971). Moreover, plants overexpressing phytochrome A or phytochrome B show delayed leaf yellowing (Rousseaux et al., 1997; Thiele et al., 1999). Leaves of *phyB* mutant are hyposensitive to dark treatment (Brouwer et al., 2014). These results imply a role of phytochromes in the regulation of leaf senescence, but the connection between phytochrome-mediated signaling pathways and the physiological mechanisms underlying the regulation of leaf senescence remains poorly understood.

Phytochromes regulate light responses by promoting the degradation of PIFs (phytochrome-interacting factors), a family of basic helix–loop–helix (bHLH) transcription factors that promote hypocotyl elongation in the dark (Lorrain et al., 2008). PIFs act as an important hub of light, auxin, gibberellic acid, and brassinosteroid pathways through the regulation of over 1000 genes’ expression by binding to G-box or E-box elements (Leivar and Quail, 2011; Hornitschek et al., 2012). Different PIFs play redundant and distinct roles in *Arabidopsis* (Jeong and Choi, 2013; Pfeiffer et al., 2014). For example, PIF3 plays a role in the chloroplast development and chlorophyll biosynthesis (Monte et al., 2004); PIF4 controls the thermosensory activation of flowering (Kumar et al., 2012); PIF7 regulates shade avoidance response (Li et al., 2012). When we found that *PIF3*, *4*, and *5* were up-regulated during age-triggered leaf senescence and dark-induced detached leaf senescence based on several published microarray data (Lin and Wu, 2004; Buchanan-Wollaston et al., 2005; van der Graaff et al., 2006; Breeze et al., 2011), we wondered whether the increased expression of the *PIFs* exerts a role in the regulation of leaf senescence and what the mechanism is.

In this study, we found that the expression level of *PIF3*, *4*, and *5* was significantly up-regulated during both age-triggered and dark-induced leaf senescence. All of the *PIF3*, *4*, and *5* single mutants exhibited significantly delayed leaf senescence, whereas their overexpressors resulted in early senescence. Moreover, transcriptome analysis identified that PIF4’s target genes were involved in chlorophyll degradation and chloroplast activity. Finally, we demonstrated that PIF4 could regulate dark-induced ethylene biosynthesis and ethylene-induced leaf senescence.

## RESULTS

### *PIF3*, *4*, and *5* Are Early Senescence Responsive Genes

**Figure 1:**
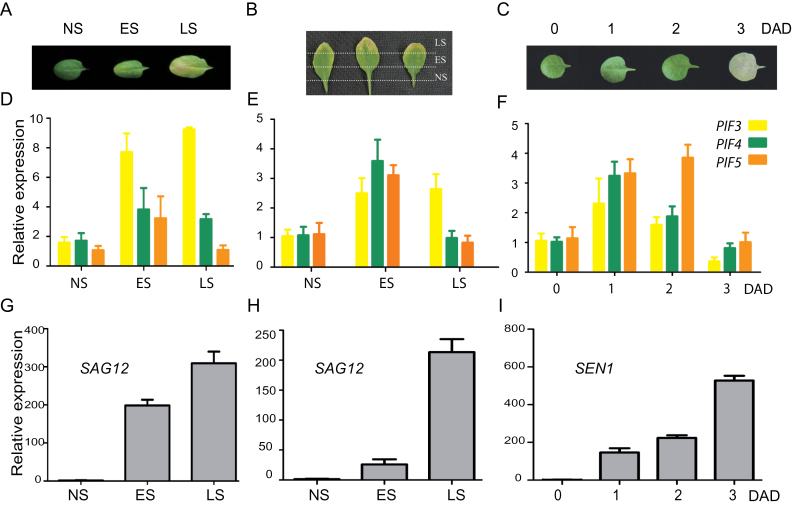
*PIF3*, *4*, and *5* Are Early Senescence Responsive Genes. **(A)** Different senescence stages of leaves from 4-week-old Col-0 plants. NS, non-senescence; ES, early senescence; LS, late senescence. **(B)** Different parts of a senescing leaf. **(C)** Phenotypes of the leaves from 4-week-old Col-0 plants and after dark treatment for indicated day(s). DAD, days after dark treatment. **(D–F)** Relative expressions of *PIF3*, *4*, and *5* genes in age-triggered (D, E) and dark-induced (F) senescing leaves of Col-0 plants. **(G–I)** Relative expressions of senescence marker genes *SAG12* or *SEN1* in age-triggered (G, H) and dark-induced (I) senescing leaves of Col-0 plants.

According to published microarray data, *PIF3*, *4*, and *5* were up-regulated during age-triggered and dark-induced leaf senescence (Lin and Wu, 2004; Buchanan-Wollaston et al., 2005; van der Graaff et al., 2006; Breeze et al., 2011). Consistently, we also found that the expression level of *PIF3*, *4*, and *5* was significantly induced at the early stage of leaf senescence (ES), with *PIF4* and *PIF5* trending to decrease at the late stage of leaf senescence (LS) (Figure 1A and 1D). Similar induction patterns of these *PIF*s’ expressions were detected across the senescence gradient of a single leaf (from the tip to the base) (Figure 1B and 1E). In the two experiments, *SAG12* was used as a senescence marker and an expected induction pattern of its expression was repeatedly observed (Figure 1G and 1H). By taking an advantage of a high-resolution temporal profiling of transcripts during *Arabidopsis* leaf senescence (Breeze et al., 2011), we obtained the expression pattern of *PIF1*, *3*, *4*, *5*, and *7* from day 19 to day 39 after sowing (Supplemental Figure 1A). In contrast to that of *PIF1* and *PIF7*, the expression level of *PIF3*, *4*, and *5* was increased around 29 days after sowing (DAS), which is considered the major switch time of gene expression during age-triggered senescence. When detached third and fourth rosette leaves from 4-week-old plants were incubated in darkness, *PIF3*, *4*, and *5* were induced at day 1 and day 2 but reduced at day 3 post treatment (Figure 1C and 1F) while *SEN1* was continued to be up-regulated (Figure 1I). These results suggest that *PIF3*, *4*, and *5* are early senescence responsive genes.

Further, we used *PIF3* and *PIF4* overexpression lines to examine their protein stabilities after dark treatment. Dark treatment usually triggers total protein degradation. We chose rubisco large submit protein (RbcL) as a reference protein and conducted a Western blotting. Although the RbcL level slightly decreased 48h after treatment in darkness, PIF3–MYC and PIF4–MYC proteins were significantly accumulated 12h after dark treatment and still gradually accumulated afterwards (Supplemental Figure 1B). Their induced transcriptions and increased protein stabilities suggest that PIF3, 4, and 5 possibly play certain roles during senescence.

### *PIF3*, *4*, and *5* Promote Age-Triggered and Dark-Induced Leaf Senescence

**Figure 2:**
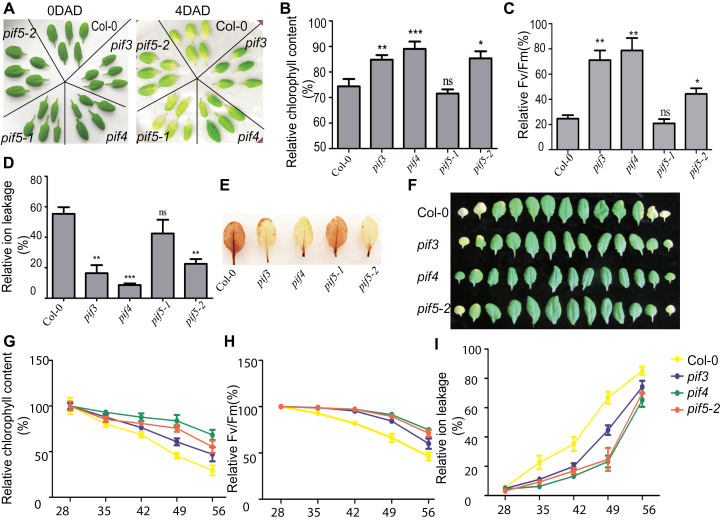
PIF3, 4, and 5 Promote Age-Triggered and Dark-Induced Leaf Senescence. **(A)** Phenotypes of dark-induced leaves from 4-week-old *pif3*, *pif4*, *pif5-1*, and *pif5-2* as well as Col-0 plants. **(B–D)** Relative chlorophyll contents (B), Fv/Fm (C), and ion leakage (D) in dark-induced leaves from 4-week-old *pif3*, *pif4*, *pif5-1*, and *pif5-2* as well as Col-0 plants. * *p* < 0.05, ** *p* < 0.01, and *** *p* < 0.001; ns, non-significant. **(E)** H_2_O_2_ levels detected by DAB staining in dark-induced leaves from 4-week-old *pif3*, *pif4*, *pif5-1*, and *pif5-2* as well as Col-0. **(F)** Phenotypes of the leaves from *pif3*, *pif4*, and *pif5-2* as well as Col-0 plants at day 42 after sowing. **(G–I)** Relative chlorophyll contents (G), Fv/Fm (H), and ion leakage (I) in the third and fourth leaves from *pif3*, *pif4*, and *pif5-2* as well as Col-0 plants at indicated days after sowing.

To determine the possible roles of PIFs in regulating senescence, we first examined the respective phenotypes of *PIF3*, *4*, and *5* mutants during dark-induced senescence. *pif3* (SALK_081927C), *pif4* (SALK_140393C) (Zhong et al., 2012), and *pif5-2* (SALK_072306) were previously reported knockout mutants, and *pif5-1* (SALK_143659) was a knockdown mutant (Khanna et al., 2007). After dark treatment, chlorophyll contents of *PIF3*, *4*, and *5* loss-of-function mutants were significantly higher than that of wild-type plants (Figure 2A and 2B). Consistently, the mutants displayed higher PSII max-photo efficiencies (Fv/Fm) and lower ion leakage (Figure 2C and 2D). Besides, we examined the expression level of two molecular markers, *SEN1* and *CAB* (Supplemental Figure 2). After dark treatment, the transcriptional induction of *SEN1* was significantly lower in the *pif* mutants than that in Col-0 and the inhibition of *CAB* transcription was decreased in *pif* mutants. DAB staining of H_2_O_2_ was used to examine the level of leaf reactive oxygen species (ROS). Loss-of-function mutants showed a decreased level of H_2_O_2_ (Figure 2E). We also examined various senescence symptoms during age-triggered senescence. It was found that the loss-of-function mutants of *pif3*, *pif4*, and *pif5-2* exhibited increased leaf longevity, as evidenced by delayed declines in chlorophyll content and Fv/Fm and delayed increases in ion leakage (Figure 2F–2I).

To confirm PIFs’ role in regulating leaf senescence, the phenotype of *PIF*s’ overexpression lines was also analyzed during both age-triggered and dark-induced senescence. The difference in the chlorophyll content between Col-0 and *PIF*s’ overexpression lines was markedly visible 42 DAS or 3 d after dark treatment (DAD), and severely yellowing leaves were observed in PIFs overexpression lines (Supplemental Figure 3). Furthermore, declines in Fv/Fm were also accelerated in these lines during both age-triggered and dark-induced senescence. These results collectively demonstrate that PIF3, PIF4, and PIF5 positively regulate both age-triggered and dark-induced leaf senescence.

### Transcriptome Analysis of *pif4* Mutant

Based on the phenotypic analysis, *pif4* knockout mutant showed the strongest delay of senescence. As such, we focused on PIF4 to explore its downstream target genes. As *PIF4* is an early senescence responsive gene, we treated leaf tissues in darkness for 24h to induce synchronous senescence. RNA samples were prepared from the third and fourth rosette leaves of 4-week-old Col-0 and *pif4* plants. The samples prepared from leaf tissues before and after dark treatment were sequenced.

**Figure 3:**
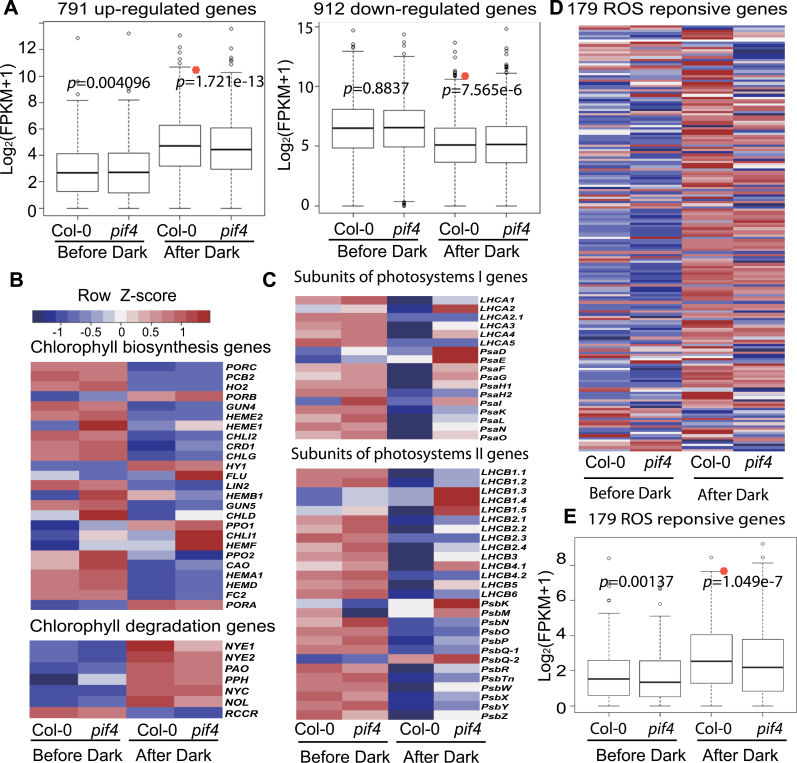
Transcriptome Analysis of *pif4* Mutant. **(A)** Box-plot representations of 791 senescence up-regulated and 912 senescence down-regulated gene expressions in *pif4* and Col-0 leaves before and after dark treatment for 24h. Paired *t*-test was used in statistical analysis. FPKMs, fragments per kilobase of exon per million fragments mapped. **(B)** Heat map showing the expression of chlorophyll biosynthesis and degradation genes in Col-0 and *pif4* leaves before and after dark treatment for 24h. **(C)** Heat map showing the expression of subunit genes of photosystems I and II in Col-0 and *pif4* leaves before and after dark treatment for 24h. **(D)** Heat map showing the expression of 179 ROS-responsive genes in Col-0 and *pif4* leaves before and after 24h dark treatment. **(E)** Box-plot representation of expressions of 179 ROS responsive genes in Col-0 and *pif4* leaves before and after dark treatment for 24h.

First, we compared a list of 791 senescence up-regulated genes and 912 senescence down-regulated genes previously reported (van der Graaff et al., 2006) during both dark-induced and age-triggered senescence to examine the effect of PIF4 on early senescence transcriptome (Supplemental Datasets 1 and 2). As shown in the box plot (Figure 3A), expression levels of 791 and 912 genes were similar between Col-0 and *pif4* before dark treatment; however, 24h after dark treatment, the expressions of these genes were significantly different (*p* = 1.721e-13 for senescence up-regulated genes and *p* = 7.565e-6 for down-regulated genes), suggesting that PIF4 plays a role in the regulation of the transcriptome during early leaf senescence.

The most striking feature of leaf senescence is the yellowing caused by the breakdown of chlorophyll. This phenomenon was delayed in *pif4*. So it is interesting to investigate whether PIF4 accelerated senescence through the regulation of biosynthesis and/or degradation of chlorophyll. The transcriptional level of several chlorophyll biosynthesis genes and degradation genes were pulled out from the dataset (Supplemental Dataset 3) and a heat map was constructed (Figure 3B). Dark treatment significantly inhibited the biosynthesis and promoted the degradation of chlorophyll, whereas *pif4* mutant exhibited an obvious reduced inhibition and promotion.

On the list of down-regulated genes, PIF4 affected photosynthesis-associated GO terms most significantly (Supplemental Dataset 4). A large number of photosynthesis-related genes were down-regulated 24h after dark treatment in Col-0 but not in *pif4*, such as *Lhcb2.1*, *Lhcb2.2*, and *Lhcb5*. According to the heat map of subunits genes of photosystems I and II (Figure 3C and Supplemental Dataset 5), it is obvious that PIF4 is involved in the negative regulation of the chloroplast activity.

Besides its function in regulating chloroplast activity, PIF4 also showed an effect on H_2_O_2_ status (Figure 2E). According to RNA-sequencing data, most of ROS responsive genes (Wu et al., 2012) were induced 24h after dark treatment in third or fourth leaves in Col-0 but the induction was attenuated in *pif4*, suggesting that PIF4 promotes the generation of ROS and the expression level of stress-related genes (Figure 3D, 3E, and Supplemental Dataset 6).

### PIF4 Negatively Regulates Chloroplast Activity

**Figure 4:**
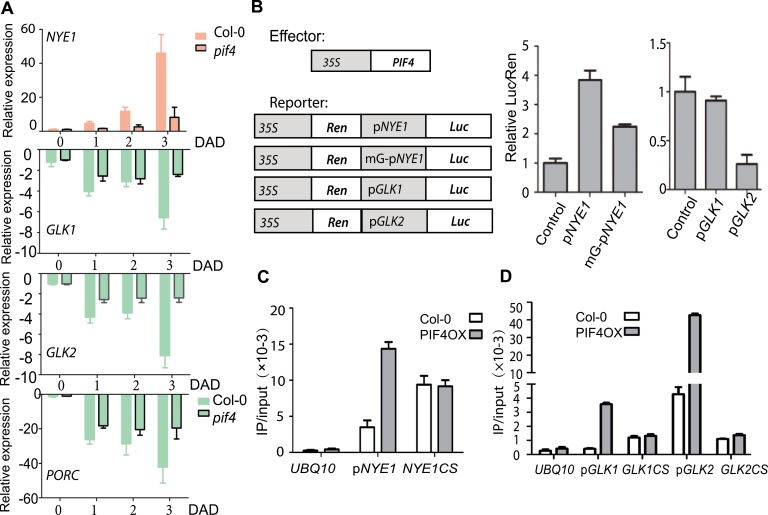
PIF4 Negatively Regulates Chloroplast Activity. **(A)** Relative expressions of *NYE1*, *GLK1*, *GLK2*, and *PORC* in dark-induced leaves from 4-week-old *pif4* and Col-0 plants. **(B)** Effects of PIF4 on the activity of *NYE1*, *GLK1*, and *GLK2* promoters in *Arabidopsis* protoplasts. Effector constructs contain the CaMV 35S promoter fused to the transcription factor PIF4. Reporter constructs contain 512-, 1505-, and 1589-bp promoters of *NYE1*, *GLK1*, and *GLK2* upstream of their translation initiation sites, respectively, with G-box or E-box element, fused to the LUC reporter gene. Effects of PIF4 on the activity of *NYE1*, *GLK1*, and *GLK2* promoter fragments were expressed as a ratio of Luc to Ren. **(C)** Direct binding of PIF4 with the promoter of *NYE1*. ChIP assays were conducted by real-time PCR after normalizing with the input DNA. The fragment of *PIF4* coding sequence (CS) was used as a negative control. **(D)** Direct bindings of PIF4 with the promoters of *GLK1* and *GLK2*. ChIP assays were conducted by real-time PCR after normalizing with the input DNA. The fragment of *PIF4* CS was used as a negative control.

Since PIF4 negatively affected photosynthesis-associated GO terms with most significant *p*-values (Supplemental Dataset 4), it is reasonable to investigate whether chlorophyll and chloroplast related genes are the targets of PIF4. We initially examined the expression of *NYE1*, *GLK1*, *GLK2*, and *PORC* by qRT–PCR (Figure 4A). *PORC* has been reported to be a direct target gene of PIF4 (Toledo-Ortiz et al., 2014) and, as expected, the expression level of *PORC* was higher in *pif4* than that in Col-0 after dark treatment.

Interestingly, qRT–PCR analysis showed that the induction of *NYE1* was largely inhibited in *pif4* mutant. By fusing *NYE1* promoter with Luciferase gene and testing its activation by overexpressing *PIF4* in *Arabidopsis* protoplasts, we found that PIF4 could dramatically increase WT *NYE1* promoter activity (Figure 4B). However, after the G-box in *NYE1* promoter region was mutated, the activation was significantly reduced (Figure 4B). PIF4 was attached in the G-box region of *NYE1* promoter based on ChIP–PCR data (Figure 4C), suggesting that PIF4 could directly regulate the transcription of *NYE1*.

Recently published PIF4 chip-sequencing data revealed that chloroplast activity maintainer genes *GLK1* and *GLK2* were PIF4’s target genes (Oh et al., 2012). *GLK1* and *GLK2* are upstream positive regulators of chlorophyll biosynthesis and chloroplast development. Overexpressing either of them causes more chlorophyll biosynthesis and enhanced leaf longevity, whereas *glk1glk2* double mutant exhibited paled phenotype (Waters et al., 2009). Our qRT–PCR result showed that both *GLK1* and *GLK2* expressions were higher in *pif4* mutant after dark treatment than those in Col-0 (Figure 4A), although there was no dramatic effect observed 1 DAD. To further confirm the negative regulation of PIF4 on the promoter of *GLK1/2*, Dual-Luciferase assay was performed in *Arabidopsis* protoplasts. It was shown that PIF4 significantly repressed *GLK2* expression but not *GLK1* expression, and the latter was proposed to be regulated by BZR1 and PIF4 synergistically (Oh et al., 2012) (Figure 4B). We further conducted a ChIP–PCR experiment and confirmed that PIF4 did bind with E-box regions of both *GLK1* and *GLK2* promoter (Figure 4D).

### PIF4 Regulates Dark-Induced Ethylene Biosynthesis and Ethylene-Induced Leaf Senescence

**Figure 5:**
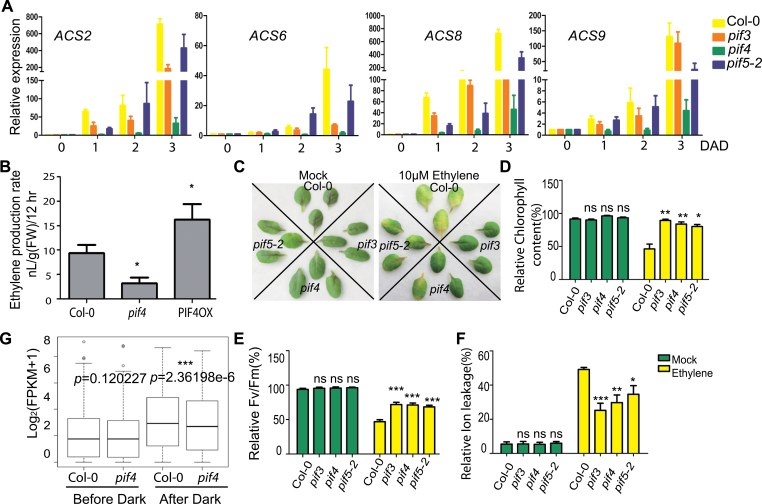
PIF4 Regulates Both Ethylene Biosynthesis and Signaling. **(A)** Relative expressions of *ACS2*, *ACS6*, *ACS8*, and *ACS9* in dark-induced leaves from Col-0 plants and *pif* mutants. **(B)** Ethylene levels in third or fourth rosette leaves from 4-week-old Col-0 and *pif4* mutant 12h after dark treatment. **(C–F)** Ethylene-induced senescence was delayed in *pif3*, *4*, and *5* mutant lines. Relative chlorophyll contents (D), relative Fv/Fm (E), and relative ion leakage (F) were measured. * *p* < 0.05, ** *p* < 0.01, and *** *p* < 0.001; ns, non-significant. (F) Box-plot representation of expressions of 164 ACC induced genes in Col-0 and *pif4* before and after dark treatment for 24h.

As a cellular signaling hub, PIFs have been reported to integrate multiple phytohormone signals to orchestrate the regulation of the transcriptional network that drives multiple facets of downstream morphogenesis (Leivar and Quail, 2011). Ethylene is an inducer of leaf senescence and dark treatment could induce ethylene production (Bleecker and Kende, 2000; van der Graaff et al., 2006). Our RNA-sequencing analysis showed that expression levels of several ethylene biosynthesis genes (*ACSs*) were up-regulated by dark treatment and PIF4. We then performed qRT–PCR to verify this result. As shown in Figure 5A, *ACS2*, *ACS6*, *ACS8*, and *ACS9* were all induced after dark treatment, but the induction dramatically decreased in *pif4* mutants and also reduced in *pif3* and *pif5* mutant to a certain extent.

To explore the relationship between PIF4 and ethylene biosynthesis, we measured the ethylene level in the third or fourth leaves of 4-week-old *pif4* plants and PIF4OX lines 12h after dark treatment. As expected, ethylene levels declined significantly in *pif4* plants while being elevated significantly in PIF4OX lines (Figure 5B), suggesting that the induction of ethylene biosynthesis by dark treatment is at least partially mediated by PIF4. Furthermore, exogenously applied ethylene could partially rescue delayed dark-induced senescence phenotype in *pif4* (Supplemental Figure 4). These results confirmed that PIF4 could promote ethylene biosynthesis. An equally intriguing issue is if PIF4 is also involved in ethylene signaling. To answer this question, we treated the third or fourth rosette leaves taken from 4-week-old Col-0 and *pif*s plants with ethylene. The result showed that *pif3*, *pif4*, and *pif5-2* significantly delayed senescence, as demonstrated by delayed declines in chlorophyll content and Fv/Fm value and delayed elevation in ion leakage (Figure 5C–5F).To confirm the effects of PIF4 on ethylene synthesis and signaling, we further examined the expression level of ethylene-responsive genes between Col-0 and *pif4* based on our RNA-sequencing data. The expression level of 164 ACC-responsive genes (Nemhauser et al., 2006) was significantly lower in *pif4* than that in Col-0 24h after dark treatment (Figure 5G and Supplemental Dataset 7), which could be caused by either reduced ethylene biosynthesis or ethylene signaling.

## DISCUSSION

**Figure 6:**
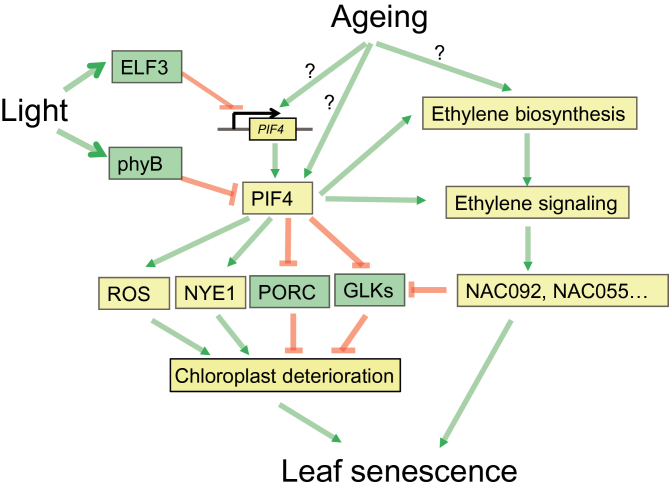
A Proposed Model for PIF4-Mediated Leaf Senescence.

In this study, we have shown that PIF3, 4, and 5 promote age-triggered and dark-induced leaf senescence based on phenotypic analyses of their mutants and overexpression lines. Identification of PIF4’s target genes prompted us to propose a working hypothesis of PIF4-dependent regulation of leaf senescence. According to this hypothesis (Figure 6), PIF4 acts as a transcriptional activator that up-regulates the expression of *NYE1* to trigger the degradation of chlorophyll and as a transcriptional repressor for *PORC* and *GLK2* to inhibit the chlorophyll biosynthesis and chloroplast activity. PIF4 also somehow stimulates ROS generation systems to promote senescence. Moreover, activation of ethylene biosynthesis and ethylene signaling reveals an additional way of PIF4’s involvement in the regulation of leaf senescence. Our finding delineated the function of PIF3, 4, and 5 on developmental and dark-induced leaf senescence, identified PIF4’s target genes which well explains delayed senescence phenotype in *pif4*, and proposed a possible mechanism by which light signaling regulates leaf senescence through the PIFs.

Consistently with our results, the transcription of *PIF4* and *5* has been reported to increase in the dark-held inflorescences and mutants defective in *PIF* expression delayed inflorescence de-greening (Trivellini et al., 2012). Glucose supplementation in immature inflorescences antagonized carbon deprivation and suppressed the dark-induced transcript accumulation of *PIF4* and *5*, which in turn delayed the de-greening process of inflorescence (Trivellini et al., 2012). In contrast, leaves, as vegetative organs, displayed a differential response to glucose treatment, namely acceleration of senescence (Wingler et al., 2006), suggesting the divergence on the metabolic program associated with senescence between these two organs. Despite this, it is consistent that PIFs are involved in the positive regulation of senescence in both inflorescences and leaves.

What is responsible for *PIF*s’ induction in dark-induced and age-triggered leaf senescence? ELF3–ELF4–LUX complex has been reported to repress the expression of *PIF4* and *5* in the evening phase (Nusinow et al., 2011), which may also regulate the transcription of *PIF4* and *5* during dark-induced leaf senescence. Paek’s and Choi’s groups confirmed the inhibition of senescence by ELF3 in darkness (Sakuraba et al., 2014). We searched the transcription level of *ELF3* from the transcriptional profiling of leaf senescence from day 19 to day 39 after sowing (Supplemental Figure 5); *ELF3* was not significantly repressed by senescence. The function of ELF3 in age-triggered leaf senescence still needs to be confirmed.

In this study, we also observed that the loss-of-function mutant of *PIF3* displayed a similar senescence phenotype as *pif4* and *pif5-2* mutants. We detected the transcriptional induction of *PIF3* during senescence by RT–PCR, which is consistent with several microarray data (Lin and Wu, 2004; Buchanan-Wollaston et al., 2005; van der Graaff et al., 2006; Breeze et al., 2011). The induction of *PIF3* starts late but lasts longer than that of *PIF4* and *PIF5* (Figure 1 and Supplemental Figure 1). Currently, we have no idea as yet how PIF3 is exactly involved in the regulation of leaf senescence. Nevertheless, one of the possibilities is that the increased ethylene level during dark- and age-triggered leaf senescence may stimulate PIF3’s expression through EIN3 (Ethylene Insensitive 3) (Zhong et al., 2012). We have shown that PIF4 is partially responsible for dark-induced ethylene biosynthesis, and it is plausible that PIF4-mediated increase of ethylene level leads to the induction of PIF3. PIF4 shares similar target genes as well as functions with other PIFs that can also play diverse roles in various processes (Jeong and Choi, 2013; Pfeiffer et al., 2014). It is therefore not surprising if PIF3 shares a partial function with PIF4, being involved in the regulation of ethylene biosynthesis. However, it would be really interesting to find out what the upstream regulatory factor(s) is and what exact causal relationship between ethylene biosynthesis/signaling and the PIFs’ function is. Unexpectedly, Sakuraba and his colleagues did not observe a delayed senescence phenotype in *pif3* mutant (Sakuraba et al., 2014). This inconsistent observation may be attributed to the different origin of the mutant lines or to the different age of leaves analyzed.

The major function of PIF4 we identified is related with chloroplast activity. Besides several chlorophyll biosynthesis genes, *NYE1* and *GLK2* were the main targets of PIF4. PIF4 could function as both an activator and a repressor. Sakuraba and colleagues found that PIF4 is involved in the regulation of ethylene-induced leaf senescence via activating the transcription of *EIN3*. This is consistent with our observation of *pif4*’s senescence phenotype upon ethylene treatment. Moreover, we found that PIF4 promoted ethylene biosynthesis. PIF4 affected the expression of ethylene-responsive genes likely via its direct regulation as *NAC092* (Sakuraba et al., 2014).

ROS are normal products of metabolism, including superoxide free radicals, H_2_O_2_, and hydroxyl radicals. It has long been considered that various senescence physiological processes are largely mediated by the dramatic increase in ROS during senescence, such as marked increases in lipid peroxidation and membrane leakage, and extensive loss of chlorophyll and proteins (Thompson and Lake, 1987). Moreover, ROS also act as signal molecules to induce many SAGs expression and accelerate leaf senescence or cell death (Navabpour et al., 2003). In our study, PIF4 was found to positively regulate ROS level during dark-induced leaf senescence. However, the intricate molecular mechanism of PIF4’s involvement in the regulation of ROS generation remains to be elucidated.

The study described in this report demonstrates the underlying molecular mechanism of phytochromes’ involvement in light regulation of leaf senescence. Our finding that the PIFs mediate leaf senescence is consistent with several reports that phytochromes act as a negative regulator of PIFs in mediating leaf senescence, such as *phyB* mutant senesces earlier as PIF overexpression lines and PHYB-OX senesces later as *pif* mutants. However, it remains to be investigated how the leaf development regulates PIFs’ transcriptions and translations, how PIF3, 4, and 5 share their targets genes, and whether this mechanism is conserved across species. Therefore, additional work is warranted for detailed mechanisms underlying how the light regulates leaf senescence.

## METHODS

### Plant Material and Growth Conditions

*Arabidopsis thaliana* plants were germinated in soil at 22ºC and grown under a long-day condition (100 μM m^–2^ s^–1^, 16-h light/8-h dark). We used the T-DNA insertion lines of *pif3* (SALK_081927C), *pif4* (SALK_140393C), *pif5-1* (SALK_143659), and *pif5-2* (SALK_072306), and PIF3OX(MYC-tag) and PIF4OX(Flash-tag) lines.

For inducing senescence of detached leaves in darkness, the third and fourth rosette leaves from 4-week-old *Arabidopsis* plants were excised and placed in Petri dishes. Petri dishes were sealed with parafilm tape, wrapped with double-layer aluminum foil, and then kept at 22ºC.

### Measurements of Chlorophyll Content, Ion Leakage, and Photochemical Efficiency

The detached leaves used for chlorophyll extraction were incubated in 95% acetone/ethanol (v/v = 2:1, 5% ddH_2_O) for 12h in darkness. Extracts were centrifuged at 12 000 *g* for 5min at 4ºC. Chlorophyll was quantified by measuring absorbance at 645 and 663nm, and chlorophyll content was calculated as a ratio of (20.23*A_645_ + 8.023*A_657_) g^–1^ fresh weight.

For measuring ion leakage, the detached leaves were incubated in de-ionized water for at least 2h (less than 10h) and the conductivities (C1) of the solutions were determined. The samples were then boiled in the same de-ionized water for 15min. After cooling, the conductivities (C2) of the solutions were measured again (Li et al., 2013). The ratios of C1:C2 were calculated as the degree of ion leakage. We used 2ml de-ionized water for one leaf measurement in 5ml centrifugal tube.

A LI-COR 6400 Photosynthesis-Fluence system was used to determine Fv/Fm according to the manufacturer’s protocol.

Three biological replicates were examined for measuring chlorophyll content and ion leakage; at least seven biological replicates were examined for measuring Fv/Fm. Error bars represent SEM. Student’s *t*-test was used to compare the statistical significance between Col-0 and PIFOX lines (* *p* < 0.05, ** *p* < 0.01, and *** *p* < 0.001).

### RNA-Sequencing and Data Analysis

Total RNA of the whole seedlings was extracted using a Trizol kit (Takara). 50bp single end RNA-sequencing was conducted using an Illuminia Hi-seq 2500 platform from Shanghai Genergy Biotech Co. Cufflinks methods (Trapnell et al., 2010) were used for determination of expression values. A gene with a cut-off value of two-fold change and *p*-value less than 0.01 was defined as a differentially expressed gene. GO enrichment analysis was conducted using the AgriGO website (http://bioinfo.cau.edu.cn/agriGO/) (Du et al., 2010).

Heatmap.2 in the ‘gplots’ package of R program was used for the construction of heat maps. The expression level of each gene was normalized by shifting the baseline of median value to zero; 791 senescence up-regulated genes and 912 senescence down-regulated genes were categorized according to the type ‘1’ or type ‘2’ marker of individual genes (van der Graaff et al., 2006). R package 3.0.2 was used to construct the box-plot graph.

### ChIP Assay

ChIP assays were performed as described previously (Saleh et al., 2008). Detached leaves from 4-week-old Col-0 and PIF4OX (35S::PIF4-Flash) plants were incubated in darkness for 24h. Flag M2 antibody (Sigma) was used for immunoprecipitation. ChIP assays were quantified by real-time PCR after normalizing with the input DNA. qPCR was performed using primers flanking the G-box or E-box sequence of *NYE1* and *GLK1*, *GLK2* promoters. Primers used for qPCR are listed in Supplemental Table 1. The coding sequence (CS) region was used as a negative control. *ACTIN2* was used as a reference gene.

### Protoplast Transformations and Dual-Luciferase Reporter Assay

*Arabidopsis* protoplasts were isolated from 4-week-old young leaves of *pif4* mutant. The assay was conducted as described (Sun et al., 2013). Plasmids were introduced into protoplasts by using the polyethylene glycol-mediated method. Firefly and Renilla luciferase were measured by using a Dual-Luciferase assay kit (Promega) and Synergy 2 multi-mode microplate (Bio–Tek, www.biotek.com/) as described previously (Hellens et al., 2005).

### Plasmid Construction

All the oligonucleotide primers and the constructs are listed in Supplemental Table 1.

### Ethylene Treatment and Quantification of Ethylene Production

Ethylene treatment was conducted as previously reported (Zhang and Wen, 2010). Ethephon was obtained from Shanghai Sangon Biotechnology Co., Ltd. Leaves were treated in air-tight containers (desiccators). The ethephon stock solution (1M) was prepared in ethanol. 86.5 μl 1M ethephon was added to 200ml 5mM Na_2_HPO_3_ in 17.3-L desiccators to create 5 μM ethylene environments, and then the cover was closed immediately; 346 μl and 865 μl were used to create 20 μM and 50 μM ethylene concentration environments, respectively. The desiccator was placed under light or in dark according to the experiment’s design. The third and fourth rosette leaves of 4-week-old plants were used for ethylene production measurement. Three leaves were incubated in a 2-ml Agilent gas chromatograph (GC) bottle after measuring total weight and then connected to the ETD300 ethylene detector system (Shanghai Zealquest Scientific Technology Co., Ltd). GC bottles were wrapped with double-layer aluminum foil and kept at 22ºC for 12h. We used the ‘continuous accumulation’ mode following the manufacturer’s instructions.

### Accession Numbers

PIF1 (AT2G20180), PIF3 (AT1G09530), PIF4 (AT2G43010), PIF5 (AT3G59060), PIF7 (AT5G61270), SAG12 (AT5G45890), SEN1 (AT3G45590), PORC (AT1G03630), ACT2 (AT3G18780), UBQ10 (AT4G05320), ACS2 (AT1G01480), ACS6 (AT4G11280), ACS8 (AT4G37770), ACS9 (AT3G49700), NYE1 (AT4G22920), GLK1 (AT2G20570), GLK2 (AT5G44190).

## SUPPLEMENTARY DATA

Supplementary Data are available at *Molecular Plant Online*.

## FUNDING

This work was funded by the School of Life Sciences of Fudan University and the State Key Laboratory of Genetic Engineering and Institute of Plant Biology, People’s Republic of China to L.L. and B.K., and by grants from the National Natural Science Foundation of China (3117021) and the Science and Technology Commission of Shanghai Municipality (13JC400900) to B.K. and L.L.

## Supplementary Material

Supplementary Data
