# Supplementary material for: Age-Triggered and Dark-Induced Leaf Senescence Require the bHLH Transcription Factors PIF3, 4, and 5
Source: Mol Plant. 2014 Oct 8;7(12):1776–87. doi: 10.1093/mp/ssu109 (PMC4261840; doi:10.1093/mp/ssu109)
Supplement: Supplementary Data [file supp_7_12_1776__index.html]

Age-Triggered and Dark-Induced Leaf Senescence Require the bHLH Transcription Factors PIF3, 4 and 5 — Age-Triggered and Dark-Induced Leaf Senescence Require the bHLH Transcription Factors PIF3, 4 and 5 — Age-Triggered and Dark-Induced Leaf Senescence Require the bHLH Transcription Factors PIF3, 4, and 5 — Supplementary Data 

# Age-Triggered and Dark-Induced Leaf Senescence Require the bHLH Transcription Factors PIF3, 4, and 5

## Supplementary Data

Data files

**Files in this Data Supplement:**

- Supplementary Data - Supplementary Data
- Supplementary Data - Supplementary Data
- Supplementary Data - Supplementary Data
- Supplementary Data - Supplementary Data
- Supplementary Data - Supplementary Data
- Supplementary Data - Supplementary Data
- Supplementary Data - Supplementary Data
- Supplementary Data - Supplementary Data
