# Supplementary material for: Age-Triggered and Dark-Induced Leaf Senescence Require the bHLH Transcription Factors PIF3, 4, and 5
Source: Mol Plant. 2014 Oct 8;7(12):1776–87. doi: 10.1093/mp/ssu109 (PMC4261840; doi:10.1093/mp/ssu109)
Supplement: Supplementary Data [file supp_ssu109_Supplementary_data.pdf]

Figure-S1

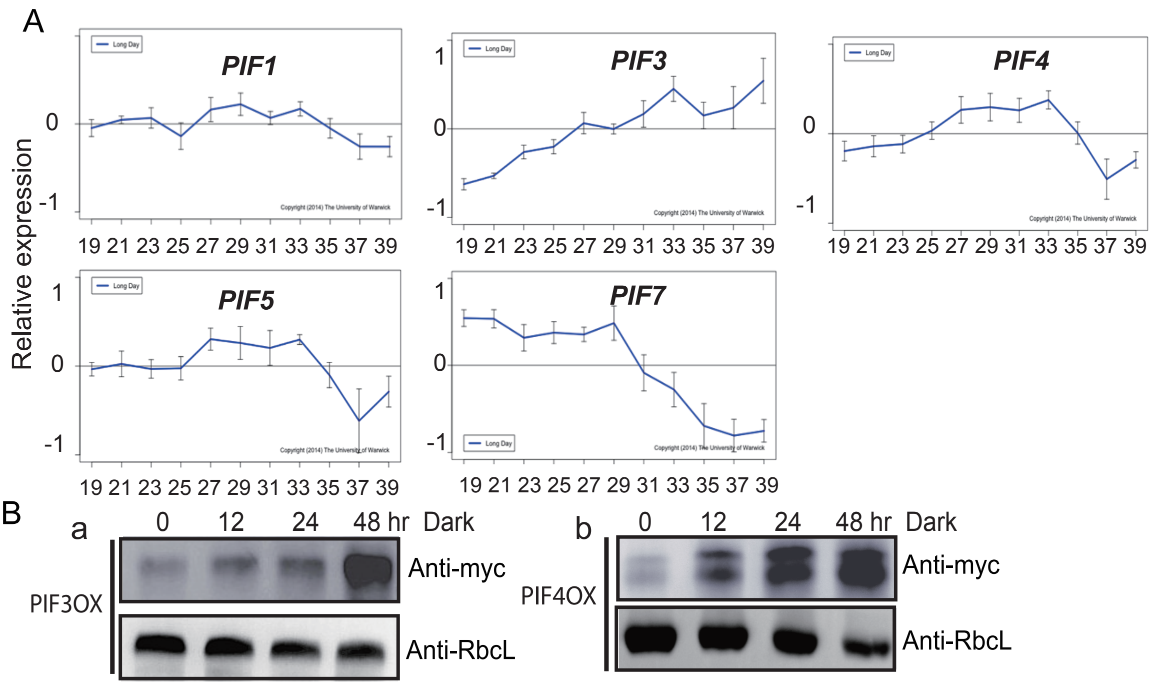

Supplemental Figure 1 Transcriptional Levels of *PIFs* During Age-Trigered Senescence and Protein Levels of PIF3 and 4 during dark-Induced Senescence.

(A) The expression of *PIFs* from 19 to 39 DAS (Days After Sowing) based on published high-resolution temporal profiling data. The graphs were generated from http://go.wawick.ac.uk/presta (Breeze, E., et al. 2011).

(B) Immunoblot analysis of PIF3,4 and Rubisco large subunit (RbcL) levels using antibodies of MYC or RbcL.Proteins were extracted from the third or fourth leaves from four-week-old PIF3OX-(a) and PIF4OX(b) plants after being incubated in darkness for 0,12,24 and 48 hr, respectively.RbcL was used as a loading control.Fresh leaves (0.1g) were collected for the assay.

Figure-S2

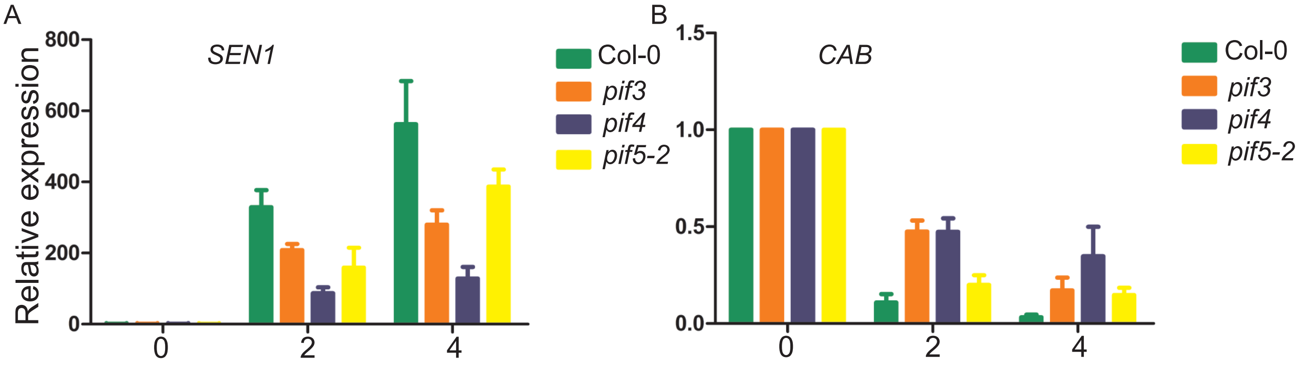

Supplemental Figure 2 The Expression of Senescence Associated Genes *SEN1* and *CAB* was Affected in *pif* Mutants During Dark Treatment.

(A) The expression level of *SEN1*,a marker gene of dark induced senescence,was dampened in indvidual *pif* mutants after dark treatment. *ACTIN2* was used as a reference gene.

(B) The expression level of *CAB*, a senescence repressed gene, was higher in respective *pif* mutants than that in Col-0 after dark treatment. *ACTIN2* was used as a reference gene.

Figure-S3

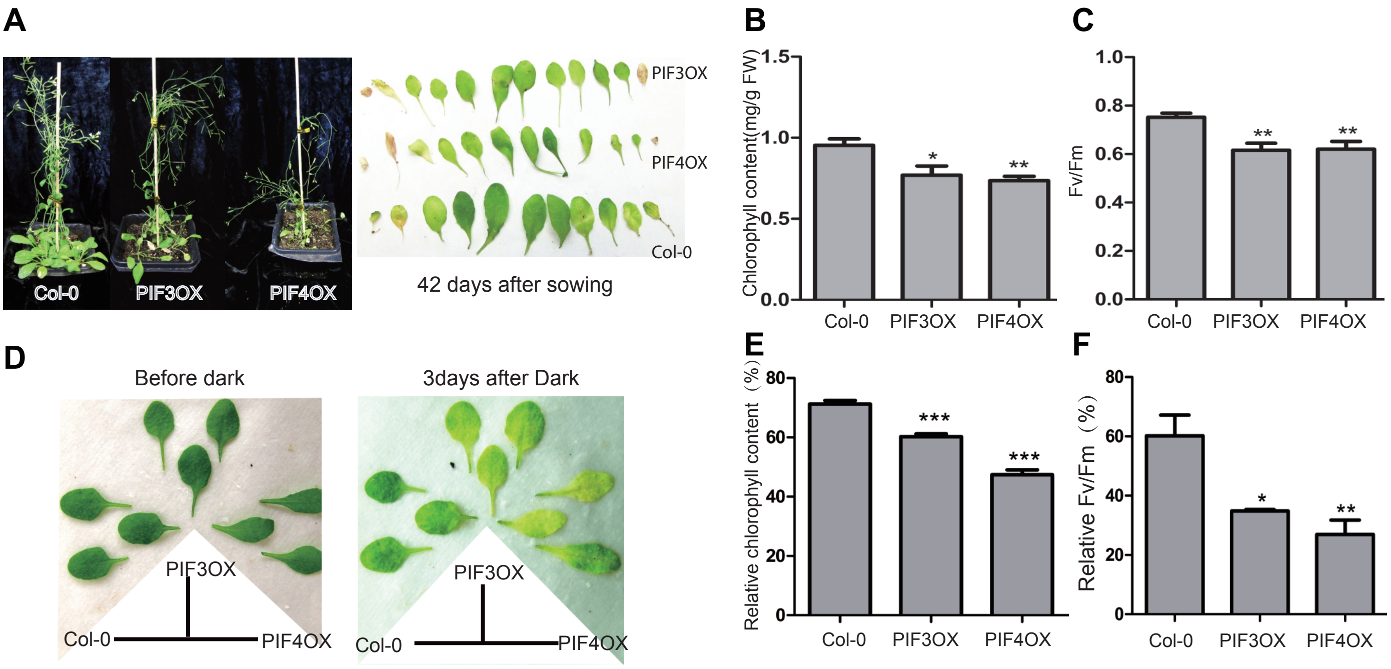

Supplemental Figure 3 PIF3OX and PIF4OX Lines Exhibited Delayed Senescence Phenotypes During Age-Triggered and Dark-Induced Senescence.

(A) Phenotypes of age-triggered senescence at day 42 after sowing.

(B-C) Chlorophyll content(B) and Fv/Fm(C) in the leaves of 42-day-old plants.The thrid and forth rosette leaves were used for above assays.*p<0.05, **p<0.01 and ***p<0.001.

(D) Phenotypes of thrid or forth rosette leaves from four-week-old Col-0, PIF3OX and PIF4OX lines 3 days after dark treatment.

(E-F) Relative chlorophyll content (E) and relative Fv/Fm (F) in the dark-induced leaves of four-week-old Col-0,PIF3OX and PIF4OX lines were measured 3 days after dark treatment.*p<0.05, **p<0.01 and ***p<0.001.

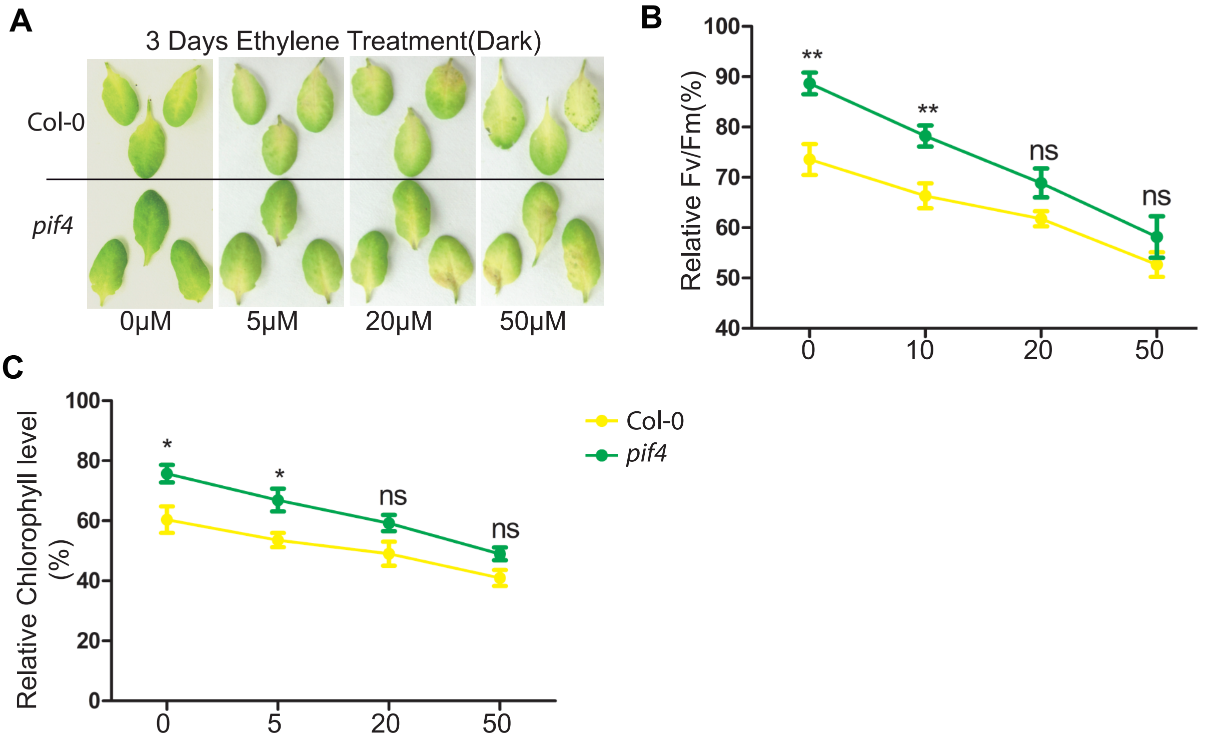

Supplemental Figure 4 PIF4 Is Involved in Dark-Induced Ethylene Biosynthesis.
(A) Ethylene partially rescued the delayed senescence phenotype of *pif4* in dosage dependent manner.
(B-C) Relative Fv/Fm and chlorophyll content in the dark-induced leaves of four-week-old Col-0 and *pif4* plants treated with ethylene were measured 4 days after dark treatment.*$p<0.05$, **$p<0.01$ and ***$p<0.001$,ns,non-significant.

Figure-S5

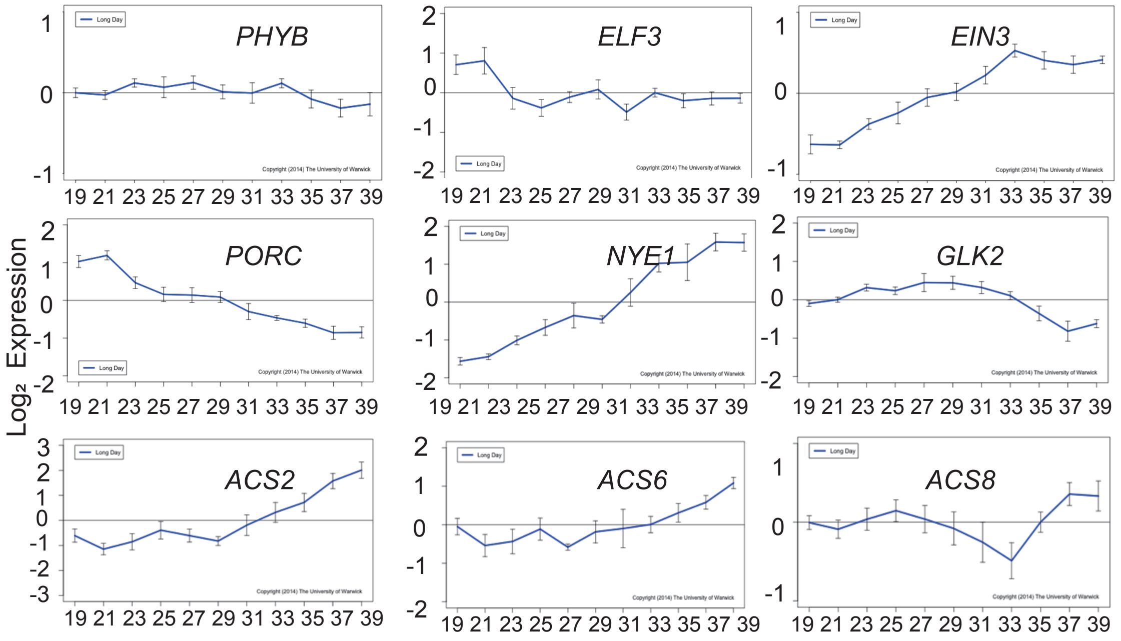

Supplemental Figure 5 The expression Level of *PHYB, ELF3, EIN3, PORC, NYE1, GLK2* and *ACSs* from day 19 to day 39 after sowing.

The analyses are based on published microarray data. The graphs were generated from http://go.wawick.ac.uk/presta (Breeze, E., et al. 2011).

Supplemental Table 1 Primers used in the experiments

	Forward primers	Reverse primers
**Primers used for RT PCR**		
PIF3	CTCGTTGACAGTAACAGGAGAC	GAATCTGCTCAAGACAGGAAC
PIF4	CGACTCAGCCGATGGAGATGTT	GTTGTTGACTTTGCTGTCCCGC
PIF5	GCGGGAAATCAGACCGTGCAACAA	CGCCGGAGATCCAAATCCCAACAT
SAG12	GCTTTCATGGCAAGACCACATAG	TGGATACGGCGAATCTACTAACG
SEN1	GTCATCGGCTATTTCTCCACCT	GTTGTCGTTGCTTTCCTCCATC
CAB	CCAGAGGCATTCGCTGAGTTG	CCTTACCAGTGACGATGGCTTG
PORC	TAAGAGGCTTAGCGTCAGGATTG	AGAGAAGCAAACGTGACTCCTGT
ACT2	CGCTCTTTCTTTCCAAGCTC	AACAGCCCTGGGAGCATC
UBQ10	AAAACCCTAACGGGAAAGACGAT	AGAACAAGATGAAGGGTGGACTC
ACS2	TCCTGGTTTTAGAGTCGGGATAGT	GCAAGCATGAGTTGTGTCTGAGA
ACS6	CTGAATCTATTGTCTAAAATCGC	ACGCATCAAATCTCCACAAAG
ACS8	ATAGGTGTCTCATGTCAACCC	GTCCAGTTTCGGTCTAATCTC
ACS9	TCGGTTTACCAGGTTTTCGC	ACACGAGTTTCTTCTGACGAA
NYE1	GCAAGGATGGGCAAATAGG	CACCGCTTATGTGACAATGAAC
GLK1/GLK2	The RT primers of GLK1 and GLK2 were the same as the following primers GLK1-CS and GLK2-CS.	
**Primer used for CHIP-PCR**		
PGLK1	AAAGTATGGTTTTTAAAGTTGCTA	ATTTGTTTACTTTTTAAGATTTTCTA
PGLK2	TCCAAAATGTAAAAAAAAATCACT	CTTGGTTGTCCCAAAAATAAAG
GLK1-CS	GACACGCAAAAGGCATATCTAT	CTAAAATGATGGTGGTGGACAG
GLK2-CS	GGTCACATCGGAAACATCTACT	TCTTCCCTCCTCCTCCTACTC
PNYE1	GTGTTGAAGGCTTTTGTGC	ACTACACTCGTCCGCACAC
NYE1-CS	GCAAGGATGGGCAAATAGG	CACCGCTTATGTGACAATGAAC
**Primers used for DNA constructs**		
PromNYE1-0800	ATAGGTACCTCGGTTAGAGCCGAATCT	ATAACTAGTCTCTGCTCTCTTGAAACC
mG-PromNYE1-0800	GATTAGAGAATTCAAAGCACTCTCTC	GAGAGAGTGCTTTGAATTCTCTAATC

PromGLK1-0800	ATAGTCGACTATGTGATACGGTAGATGGATTG	ATAGGATCCCGATCAATCTTCACTTGTAGATC
PromGLK2-0800	ATAGTCGACAGAAAAACCAAGGAGAAAACAAA	ATAGGATCCCGGAATCGTAAAAAAATGAAAA
PIF4-pCHF3	ATAGGTACCATGGAACACCAAGGTTGGAG	ATAGTCGACCTAGTGGTCCAAACGAGAACC
